# Development of Novel Microsatellite Markers in the Omei Treefrog (*Rhacophorus omeimontis*)

**DOI:** 10.3390/ijms13010552

**Published:** 2012-01-05

**Authors:** Mian Zhao, Ruiping Zhang, Chenliang Li, Taiyang Mu, Shichao Wei, Xiong Li, Hua Wu

**Affiliations:** Animal Behaviour Research Group, College of Life Sciences, Central China Normal University, 152 Luoyulu, Hongshan District, Wuhan 430079, China; E-Mails: mianzhao@yahoo.cn (M.Z.); zrp_0420@163.com (R.Z.); heibao_1986@126.com (C.L.); 1014.2mutaiyang@163.com (T.M.); weisc24@hotmail.com (S.W.); 892813767@qq.com (X.L.)

**Keywords:** Omei treefrog (*Rhacophorus omeimontis*), microsatellite markers, heterozygosity, mate choice

## Abstract

Eleven novel microsatellite markers were developed and characterized for the Omei treefrog (*Rhacophorus omeimontis*) using the fast isolation by AFLP of sequences containing repeats method. Polymorphism of each locus was tested in 24 individuals from two wild populations. The number of alleles per locus ranged from 4 to 15, the average observed and expected heterozygosity per locus ranged from 0.250 to 0.839 and from 0.562 to 0.914, respectively. Two of the 11 microsatellite loci showed significant deviations from Hardy-Weinberg equilibrium. Two locus pairs showed significant linkage disequilibrium. Neither evidence of scoring error due to stuttering nor evidence of large allele dropout was found at all of the 11 loci, but evidence of null alleles was indicated at two loci because of general excess of homozygotes for most allele size classes. These polymorphic loci will be useful markers in studying mate choice of the Omei treefrog.

## 1. Introduction

The Omei treefrog (*Rhacophorus omeimontis*), a species of amphibian in the Rhacophoridae family, is endemic to the subtropical forests in south-west China [1,2]. It has a polyandrous mating system in which females mate with more than one male per breeding season [3]. Such a reproductive form makes the Omei treefrog an excellent model for studying the evolution mechanisms of mate choice. However, little information about the mate choice of the Omei treefrog is available, mainly because from the mere traditional field work of behavior observation it is hard to quantify the mating and fertilization success of the cryptic treefrog. Fortunately, microsatellite markers not only enable the detailed analysis of mating and fertilization success of different males and females [4,5], but also enhance the feasibility of elucidating the evolution mechanisms of mate choice in the Omei treefrog. Thus, this study describes the development of 11 polymorphic microsatellite markers using the fast isolation by AFLP of sequences containing repeats (FIASCO) method, which lays a preliminary groundwork for the interesting research to uncover the evolution mechanisms of mate choice in the Omei treefrog.

## 2. Results and Discussion

Among 654 positive clones sequenced, 168 clones were found to contain repeat motifs of microsatellites, and 87 primer pairs were designed from the sequences with enough flanking region. Among the 87 primer pairs analyzed, 47 primer pairs gave consistent amplification, and were polymorphic, but only 11 primer pairs could be scored exactly (Table 1). The number of alleles per locus ranged from 4 to 15, and the average observed and expected heterozygosity per locus ranged from 0.250 to 0.839 and from 0.562 to 0.914, respectively (Table 1). After Bonferroni corrections, two of the 11 microsatellite loci (OMTF2 and OMTF8) showed significant deviations from Hardy-Weinberg equilibrium, while significant linkage disequilibrium was found between two locus pairs (OMTF4 *vs*. OMTF5, OMTF6 *vs*. OMTF8). The results of the Micro-Checker testing showed that neither evidence of scoring error due to stuttering nor evidence of large allele dropout was found at all of the 11 loci, but evidence of null alleles was indicated at two loci (OMTF2 and OMTF8) because of general excess of homozygotes for most allele size classes, which illuminated the homozygotes excess at these loci was responsible for the departure from Hardy-Weinberg equilibrium. Except the three loci (OMTF2, OMTF5 and OMTF8) that probably cause false exclusions in paternity and identity analyses [6], the probability of paternity exclusion and genetic identity was calculated for the other eight loci. The potential of paternity exclusion was greater than 99% and non-exclusion probability of genetic identity was 1.96 × 10^−11^ (Table 2), indicating that the power of these loci for parentage testing are adequately sensitive. These polymorphic loci will be useful markers in studying mate choice of the Omei treefrog.

## 3. Experimental Section

The microsatellite loci were developed using the fast isolation by AFLP of sequences containing repeats (FIASCO) method [7]. In brief, genomic DNA was extracted from muscle tissue samples of six individuals of *R. omeimontis* using a TIANamp Genomic DNA Kit (TIANGEN), and then was simultaneously digested with *Mse*I (TaKaRa) and ligated to *Mse*I AFLP adaptor (5′-GACGATGAGTCCTGAG-3′/5′-TACTCAGGACTCAT-3′) using T4 DNA ligase (Promega). The digestion-ligation mixture was diluted and directly amplified with AFLP adaptor-specific primers (5′-GATGAGTCCTGAGTAAN-3′). The amplified DNA was enriched by streptavidincoated magnetic beads (Promega) with (AGAT)_8_ and (CA)_20_ biotin-labeled probes. The enriched DNA was ligated into pMD19-T vector (TaKaRa) and transformed into competent Top10 cells. Then cells were plated onto LB agar plates containing ampicillin, IPTG and X-gal. The positive clones were identified by PCR methods using *Mse*I primer and M13 universal primer [7]. Approximately 4124 colonies were screened by PCR amplification, and 654 positive clones were sequenced on an ABI PRISM 3730 DNA Analyzer (Applied Biosystems). The obtained sequences were edited using the DNAstar software.

168 sequences containing repeat motifs of microsatellites and enough flanking region were selected for primer design using PRIMER3 [8]. Unlabelled 87 primers were initially tested and optimized on an Applied Biosystems GeneAmp 9700. Individual amplifications were performed in 10 μL reactions, containing approximately 25 ng of template DNA, 0.2 mM of each dNTP, 0.15 μM of each primer (forward and reverse), 1.5 mM Mg^2+^, 1× PCR buffer and 0.15 U of *Taq* DNA polymerase (TaKaRa). The following PCR amplification conditions were used: an initial denaturation of 95 °C for 5 min, 35 cycles of 94 °C for 30 s, annealing temperature for 30 s and 72 °C for 45 s, followed by a final extension of 10 min at 72 °C. A total of 47 primer pairs amplified consistently, and were selected for further screening. The 5′ ends of the forward primers were labeled with one of the fluorescent dye (FAM, TAMRA or HEX) for polymorphism detection. PCR reactions were repeated using the optimal annealing temperatures on an Applied Biosystems GeneAmp 9700.

Microsatellite polymorphism test was performed in 24 individuals from two populations in Sangzhi County of Hunan province and Baoxing County of Sichuan province, China. Genotyping of the 47 microsatellite loci for each individual was conducted with an ABI Prism 3730 Genetic Analyser (Applied Biosystems), and loci were scored using a GeneScan ROX 400 size standard in GeneMapper version 4.0 software (Applied Biosystems). Observed and expected heterozygosities were calculated using Arlequin version 3.5 [9]. Hardy-Weinberg equilibrium and linkage disequilibrium for each locus were assessed using Genepop version 4.0 [10]. The significant criteria for multiple simultaneous tests were adjusted using Bonferroni correction [11]. The evidence of null alleles, scoring error due to stuttering, and large allele drop out for each locus were tested using Micro-Checker version 2.2.3 [12]. The polymorphic information content and the genetic identity and paternity exclusion probabilities for each polymorphic locus and overall loci were estimated using Cervus version 3.0 [13].

## 4. Conclusions

Eleven polymorphic microsatellite markers were developed in this study, and they should be useful for further studies on mate choice in the Omei treefrog. The advances in molecular techniques will improve our understanding of the evolution mechanisms of mate choice in this species or related species.

## Figures and Tables

**Table 1:** Characteristics of eleven polymorphic microsatellite loci in the Omei treefrog (*Rhacophorus omeimontis*). *T*_a_, annealing temperature; *A*, number of alleles; *H*_O_, observed heterozygosity; *H*_E_, expected heterozygosity.

Locus	Primer sequence (5′-3′) (F, forward; R, reverse)	Repeat motif	Labelling dye	*T*_a_ (°C)	*A*	Size range (bp)	*H*_O_	*H*_E_	GenBank Accession no.
OMTF1	F:CAGGGTGACATCATAGACAA R:CTTCCAGCCACTAACAGAGC	(TG)_13_	FAM	60	5	220–236	0.542	0.631	JQ031742
OMTF2	F: CCAAGCGATGTCCCAATC R: CTCCGTGTCCACCCTGAA	(CA)_32_	TAMRE	62	14	199–255	0.458	0.914	JQ031743
OMTF3	F:AAAACACTATCATATCACCATC R: AAAATCTCCAAAAGGCAT	(AGAT)_3_ GCAT(AGAT)_4_	FAM	54	7	108–128	0.583	0.642	JQ031744
OMTF4	F: GCTGGGCTGATCCTACTCTT R: TTTGCTCTGGTACGCCTATT	(AGAT)_16_	TAMRA	60	10	243–283	0.833	0.888	JQ031745
OMTF5	F: ATTCAGCCATTCAGGAGAC R: CTGGTACGCCTATTAGTTT	(AGAT)_16_	HEX	54	10	192–232	0.839	0.902	JQ031746
OMTF6	F: AGCCATCAGATAGTTCAGG R: AATACTGCCCAAGTCAAAC	(TCTA)_18_	FAM	60	11	96–160	0.791	0.852	JQ031747
OMTF7	F: GTTTGGCTTTCAGTTAGA R: CTGGGAAGTTTGAGTTAGTT	(TCTA)_11_	HEX	53	13	168–232	0.767	0.887	JQ031748
OMTF8	F:GTGGTCAGCCAAGGAAACATAA R:AGCACCCCTAAAATGGTAGGAT	(TCTA)_15_	TAMRA	50	4	282–294	0.250	0.562	JQ031749
OMTF9	F: TGGATGAACGAGTTTGGT R: CTCCATGAGTCCAAGTGC	(AGAT)_19_	HEX	56	11	168–220	0.808	0.910	JQ031750
OMTF10	F: GCCTGGGGCAATGGATAC R: ACAACCACGAGGGAGCAA	(AGAT)_15_	TAMRA	63	15	204–280	0.825	0.895	JQ031751
OMTF11	F: GCCAGGAGCAGAATAATG R: GAGCAAACCCCACTTTGA	(TCTA)_10_	FAM	56	12	156–216	0.833	0.898	JQ031752

**Table 2:** The values of polymorphism information content (PIC) and non-exclusion probability (NE) of paternity and identity for the eight microsatellite loci in the Omei treefrog (*Rhacophorus omeimontis*). NE-1P, NE of first parent; NE-2P, NE of second parent; NE-PP, NE of parent pair; NE-I, NE of identity; NE-SI, NE of sib identity.

Locus	PIC	NE-1P	NE-2P	NE-PP	NE-I	NE-SI
OMTF1	0.574	0.786	0.615	0.430	0.190	0.488
OMTF3	0.559	0.790	0.644	0.481	0.207	0.488
OMTF4	0.859	0.405	0.252	0.092	0.028	0.322
OMTF6	0.820	0.477	0.309	0.129	0.042	0.343
OMTF7	0.855	0.415	0.260	0.100	0.030	0.323
OMTF9	0.883	0.348	0.211	0.067	0.020	0.309
OMTF10	0.865	0.394	0.245	0.090	0.027	0.318
OMTF11	0.868	0.392	0.243	0.090	0.026	0.317
Combined	0.785	2.68 × 10^−3^	1.01 × 10^−4^	1.30 × 10^−7^	1.96 × 10^−11^	2.66 × 10^−4^
